# Educational

**Published:** 1888-06

**Authors:** 


### EDITORIAL.

### Educational.

At a recent meeting of the Regents of the University of Michigan, the requirements for graduation in the department of *Dental and Oral Surgery* were increased, so that after the session of 1888 and '89 three terms of nine months each will be required for graduation. Three years ago the term was increased from six to nine months, two terms being required for graduation. Three year's study of Dentistry then as now was the requirement also. This increase of time for the course will give the student far better opportunity for a thorough mastery of the work. It will also enable the faculty to much better adjust the curriculum. A strictly graded course of instruction will be adopted, and assuredly if faithful work is done by the teachers and by the pupils, more thorough and systematic work will be accomplished by the students. The question has been asked, "Is the profession ready for a step of this kind?" The same question was asked when the term was lengthened to nine months, but that apparently made no change, for the increase in attendance continued from year to year as before, only a little larger. It is reasonable to expect that those who desire to make most thorough preparation for their life work will seek the opportunities offered for the attainment of their object. It is a matter of great regret that all of our dental colleges do not take measures for greater efficiency in their work than heretofore. Too often does the aim seem to be to secure the largest number of students without much reference to the outcome. This is greatly to be regretted, for in such a struggle the greatest disaster falls upon the student. The question should be, in the direction of competition, "who can make the most thorough work with the student, and best prepare him for his professional career."

